# A complex case of diphtheria in a pediatric patient: complications of renal failure and myocarditis

**DOI:** 10.1186/s12245-026-01146-9

**Published:** 2026-02-24

**Authors:** Abdullahi Ahmed Ahmed, Nasteha Mohamad Sheikh Omar, Ahmed Elmi Abdi, Idil Hirsi Ali, Farah Ali Ahmed

**Affiliations:** 1 Department of Emergency Medicine, Mogadishu Somali Turkey Training and Research Hospital, Mogadıshu, Somalia; 2 Department of Cardiology, Mogadishu Somali Turkey Training and Research Hospital, Mogadıshu, Somalia; 3 Department of Paediatrics, Mogadishu Somali Turkey Training and Research Hospital, Mogadıshu, Somalia; 4 Mogadishu Somali Turkey Training and Research Hospital, Mogadishu, Somalia; 5 Emergency Medicine and Critical Care Specialist, Başakşehir Çam and Sakura City Hospital (Başakşehir Çam ve Sakura Şehir Hastanesi), Istanbul, Türkiye

**Keywords:** Diphtheria, Corynebacterium diphtheriae, Myocarditis, Acute kidney injury, Antitoxin, Vaccination, Pediatric infection, Case report

## Abstract

**Background:**

Diphtheria remains a life-threatening infectious disease in low- and middle-income countries, particularly where immunization coverage is incomplete. Severe complications such as myocarditis and acute kidney injury significantly increase mortality. Reporting rare and complex presentations is essential to improve early recognition and management.

**Case presentation:**

We report the case of a 6-year-old girl who presented to the emergency department with five days of aphonia, odynophagia, progressive dysphagia, fever, confusion, and oliguria. On examination, she had cervical lymphadenopathy, pharyngeal pseudomembranes, and signs of systemic toxicity. Laboratory investigations demonstrated acute kidney injury with metabolic acidosis. Echocardiography revealed myocarditis with reduced ventricular function. The patient had **incomplete vaccination** against diphtheria. She was treated with diphtheria antitoxin, antibiotics, and supportive critical care. Despite aggressive management, the case illustrates the severe systemic complications of toxic diphtheria.

**Discussion:**

Diphtheria is transmitted primarily via respiratory droplets and direct contact with infected lesions. Children in settings with poor vaccine coverage are particularly vulnerable to severe disease. Myocarditis and renal failure are toxin-mediated complications and are the leading causes of death. This case highlights the importance of early diagnosis, laboratory confirmation, and immediate administration of antitoxin.

**Conclusion:**

This case underscores the ongoing threat of diphtheria in under-immunized populations. Prompt recognition and treatment are essential to prevent fatal complications. Strengthening routine immunization programs remains the most effective strategy to reduce diphtheria-related morbidity and mortality.

## Introduction

Diphtheria is an acute, toxin-mediated infectious disease caused by *Corynebacterium diphtheriae* that primarily affects the upper respiratory tract and, less commonly, the skin. Transmission occurs through respiratory droplets or direct contact with infected lesions, and asymptomatic carriage can contribute to continued spread of the organism [1]. Clinically, diphtheria ranges from mild pharyngitis to severe systemic illness characterized by pseudomembrane formation, cervical lymphadenopathy (“bull neck”), and multiorgan toxicity [2].

Despite the availability of effective vaccines, diphtheria remains a public health concern in parts of Africa where routine immunization coverage is incomplete and access to healthcare is limited [3]. Children in low-resource settings are particularly vulnerable to severe disease and its complications. The diphtheria toxin can cause life-threatening myocarditis, acute kidney injury, and disseminated intravascular coagulation. Among these, myocarditis is the leading cause of death, occurring in approximately 10–20% of cases and associated with mortality rates approaching 50% [4].

Outbreaks and sporadic cases of diphtheria continue to be reported in East Africa, particularly in populations affected by displacement, malnutrition, and gaps in childhood vaccination programs [5]. Delayed presentation to healthcare facilities and limited availability of diphtheria antitoxin and intensive supportive care further increase the risk of fatal outcomes in this region.

The objective of this report is to describe a complex pediatric case of respiratory diphtheria complicated by myocarditis and acute renal failure from Somalia. This case highlights the clinical severity of toxic diphtheria in a low-resource setting and underscores the importance of early diagnosis, laboratory confirmation, prompt administration of antitoxin and antibiotics, and complete immunization in preventing life-threatening complications [6].

## Case presentation

A 6-year-old girl was brought to the Emergency Department of our hospital with a 5-day history of oliguria and progressive confusion. Her illness began with aphonia, odynophagia, and a worsening sore throat, followed by intermittent fever for two days. She also complained of abdominal pain and increasing difficulty swallowing both solids and liquids. According to her caregivers, the child had **incomplete vaccination**, having missed scheduled booster doses.

She had no previous history of chronic illness, including diabetes mellitus or hypertension. Her antenatal and perinatal history were unremarkable, and she was born at term via spontaneous vaginal delivery. Her nutritional status was appropriate for age.

On admission, the patient was conscious but lethargic. Her weight was 20 kg. Vital signs showed hypotension and tachycardia: blood pressure 81/46 mmHg, pulse 136 beats/min, respiratory rate 24 breaths/min, temperature 36.4 °C, oxygen saturation 96% on room air, and random blood glucose 114 mg/dL. Capillary refill time was normal, and her extremities were warm.

Oropharyngeal examination revealed an edematous and erythematous pharynx with a thick, white pseudomembrane over the tonsils. She had marked cervical lymphadenopathy with a classic “bull neck” appearance. There was no cyanosis or respiratory distress. Chest examination showed bilateral vesicular breath sounds without wheeze or crackles. Cardiac examination revealed an irregular rhythm with a gallop sound and no audible murmur.

**Fig. 1:**
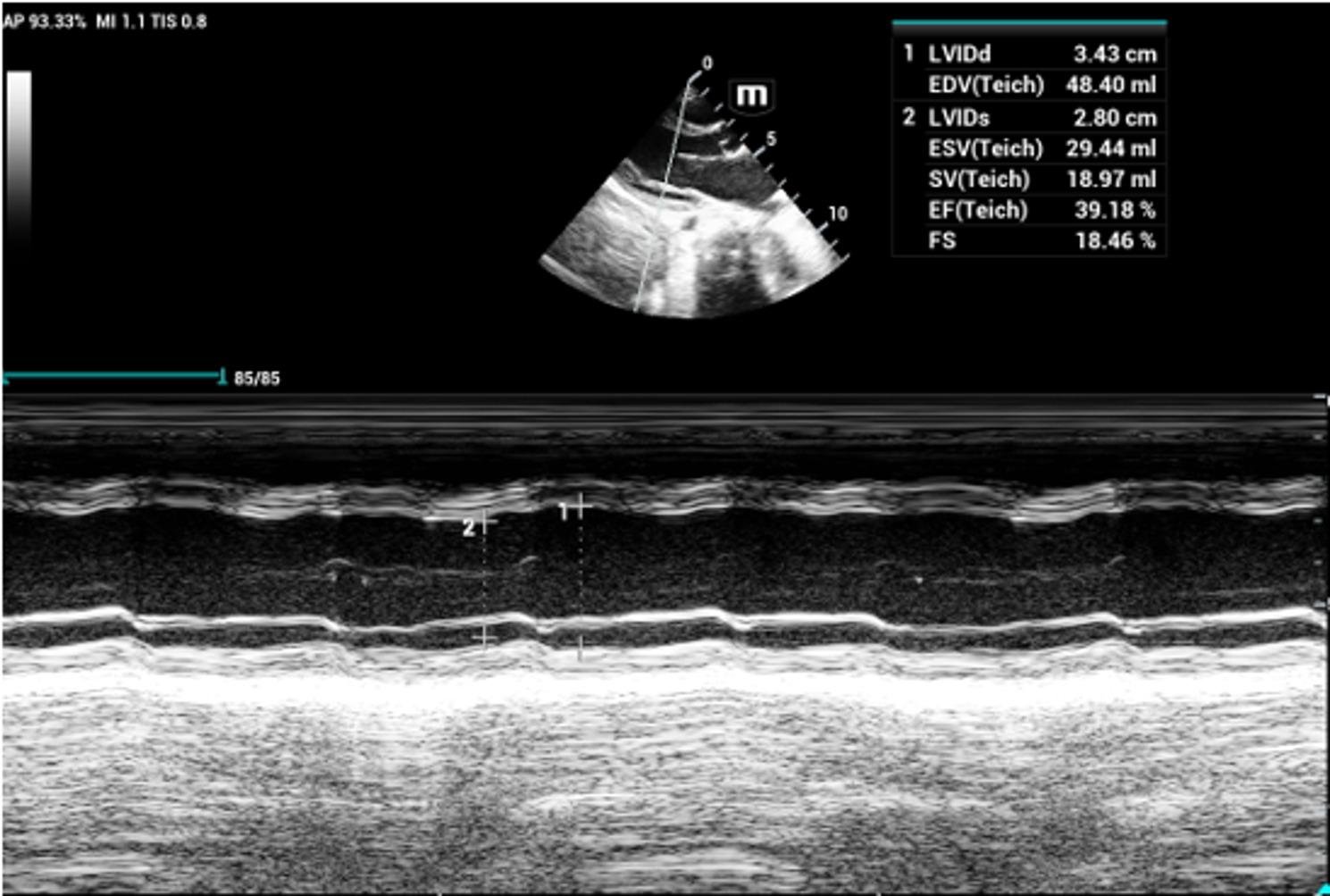
Transthoracic echocardiography with M-mode: showing of impaired left ventricular systolic function

**Fig. 2:**
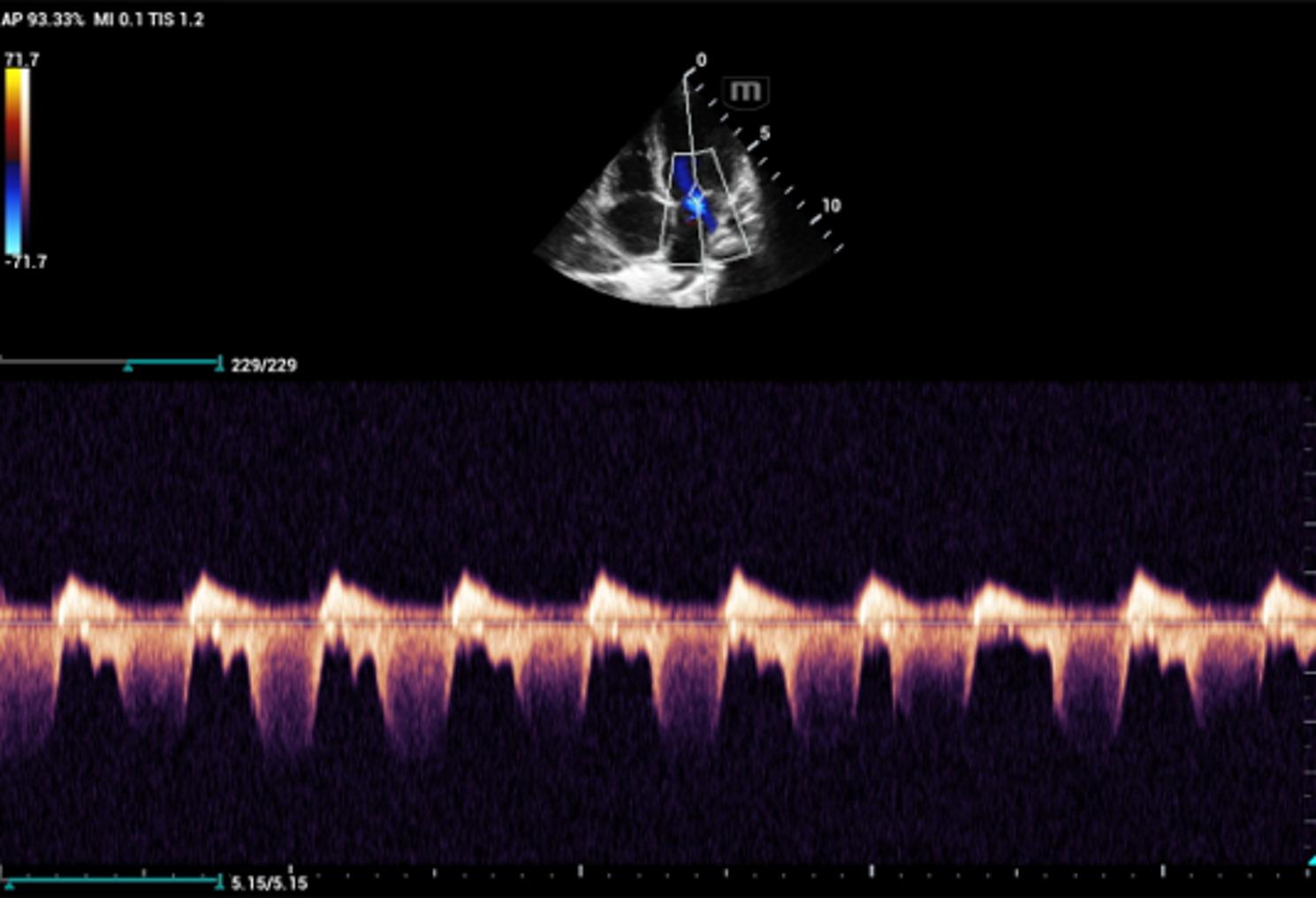
Transthoracic echocardiography (**Four-Chamber View**): demonstrates functional mitral valve regurgitation secondary to myocarditis

Transthoracic echocardiography demonstrated impaired left ventricular systolic function on M-mode imaging (Fig. 1) and functional mitral regurgitation on the four-chamber view, consistent with **acute myocarditis and heart failure** (Fig. 2). Chest radiography showed cardiomegaly without pulmonary infiltrates. Abdominal examination was soft and non-tender with normal bowel sounds.

Based on the presence of pseudomembrane, cervical lymphadenopathy (“bull neck”), and systemic toxicity, a diagnosis of **toxic respiratory diphtheria** was made.

Laboratory investigations revealed severe **acute kidney injury**, with markedly elevated urea and creatinine levels, along with electrolyte disturbances including hyperkalemia, hypocalcemia, and hyperphosphatemia. Arterial blood gas analysis demonstrated metabolic acidosis. C-reactive protein was markedly elevated, indicating severe inflammation. Complete blood count showed severe anemia, thrombocytopenia, leukopenia, and anisocytosis. Liver enzymes showed mildly elevated AST with normal ALT. Urinalysis was consistent with renal involvement and urinary tract infection. Viral serologies for HBsAg, anti-HCV, and anti-HIV were negative.

The patient was admitted for urgent management. She received intravenous fluids (normal saline with 5% dextrose, 1500 mL/24 h), intravenous paracetamol, and omeprazole. Specific therapy included **anti-diphtheria serum (60**,**000 IU)** and intramuscular procaine penicillin (700,000 IU daily for 10 days). Supportive treatment consisted of cetirizine, sucralfate, multivitamins, and nutritional support with soft diet and milk feeds. Renal and cardiac functions were closely monitored.

This case represents severe **toxic diphtheria complicated by myocarditis and acute renal failure in a child with incomplete immunization**, illustrating the life-threatening nature of the disease in low-resource settings.

## Discussion

Diphtheria is a vaccine-preventable disease, yet it continues to cause significant morbidity and mortality in low- and middle-income countries where immunization coverage is incomplete and health-care access is limited. In East Africa, recurrent outbreaks and sporadic cases have been reported, particularly in settings affected by displacement, malnutrition, and gaps in routine childhood vaccination programs [7–9]. Somalia, like several countries in the region, faces persistent challenges in achieving full DTP vaccine coverage, creating conditions for the re-emergence of toxic diphtheria [9, 10].

Our patient presented with classic features of toxic respiratory diphtheria, including pseudomembrane formation, cervical lymphadenopathy (“bull neck”), and systemic toxicity. The diagnosis was further supported by echocardiographic evidence of myocarditis and laboratory findings consistent with acute kidney injury and severe inflammation. These findings meet the clinical case definition of diphtheria and illustrate the multisystem effects of diphtheria toxin [11].

The most serious complications of diphtheria are myocarditis, neuritis, and nephritis. Myocarditis is the leading cause of death, occurring in approximately 10–20% of patients and associated with mortality rates approaching 50% [12]. In our case, the child developed acute myocarditis with impaired left ventricular systolic function and functional mitral regurgitation, highlighting the cardiotoxic effects of diphtheria toxin. The presence of an irregular rhythm and gallop rhythm further supported myocardial involvement [12, 13].

Acute kidney injury in diphtheria is less frequently reported but represents a severe manifestation of systemic toxin dissemination. The markedly elevated urea and creatinine levels, electrolyte disturbances, and metabolic acidosis in this patient are consistent with toxin-mediated nephritis and renal hypoperfusion secondary to cardiac dysfunction [13]. The combination of myocarditis and renal failure significantly increases the risk of mortality and reflects advanced disease at presentation [13, 14].

Incomplete immunization was a critical factor in this case. Although vaccines are highly effective at preventing clinical disease, they do not always prevent colonization. However, unvaccinated or partially vaccinated children are at much higher risk of developing severe toxin-mediated illness [7, 10]. In East Africa, missed booster doses and disrupted immunization schedules remain common, especially in conflict-affected and resource-limited settings [9, 10]. This case underscores the vulnerability of children who do not complete the full diphtheria vaccination series.

Early administration of anti-diphtheria serum (ADS) is the cornerstone of treatment, as it neutralizes circulating toxin but does not reverse tissue damage already established [11, 12]. Therefore, delayed presentation—as occurred in this patient—reduces the effectiveness of antitoxin therapy and increases the likelihood of cardiac and renal complications [12].

Antibiotics such as penicillin or erythromycin are essential to eradicate the organism and prevent further toxin production and transmission [11, 12]. Supportive care plays a major role in survival, including hemodynamic stabilization, correction of electrolyte and acid–base disturbances, nutritional support, and close monitoring of cardiac and renal function. In low-resource settings, limited access to intensive care and dialysis further worsens outcomes in patients with advanced diphtheria [9, 14].

## Conclusion

This case illustrates the severe and life-threatening complications of toxic diphtheria in a child with incomplete immunization. The patient developed multisystem involvement, including myocarditis and acute kidney injury, reflecting advanced toxin-mediated disease at presentation. Delayed care and lack of full vaccination likely contributed to disease severity.

Early recognition of classical clinical features—pseudomembrane, cervical lymphadenopathy, and systemic toxicity—combined with prompt administration of anti-diphtheria serum and appropriate antibiotics are essential to reduce morbidity and mortality. However, antitoxin therapy cannot reverse established organ damage, emphasizing the importance of early diagnosis and referral.

This case highlights the continued risk of diphtheria in low-resource and under-immunized settings such as East Africa. Strengthening routine childhood.

## Data Availability

The data that support the findings of this study are available in Mogadishu Somali Turkey, Recep Tayyip Erdogan Training and Research Hospital information system. Data are however allowed to the authors upon reasonable request and with permission of the education and research committee.
